# Value of Engagement in Digital Health Technology Research: Evidence Across 6 Unique Cohort Studies

**DOI:** 10.2196/57827

**Published:** 2024-09-03

**Authors:** Sarah M Goodday, Emma Karlin, Alexa Brooks, Carol Chapman, Christiana Harry, Nelly Lugo, Shannon Peabody, Shazia Rangwala, Ella Swanson, Jonell Tempero, Robin Yang, Daniel R Karlin, Ron Rabinowicz, David Malkin, Simon Travis, Alissa Walsh, Robert P Hirten, Bruce E Sands, Chetan Bettegowda, Matthias Holdhoff, Jessica Wollett, Kelly Szajna, Kallan Dirmeyer, Anna Dodd, Shawn Hutchinson, Stephanie Ramotar, Robert C Grant, Adrien Boch, Mackenzie Wildman, Stephen H Friend

**Affiliations:** 1 4YouandMe Seattle, WA United States; 2 Department of Psychiatry University of Oxford Oxford United Kingdom; 3 Crohn's & Colitis Foundation New York, NY United States; 4 Section of Urology and Renal Transplantation Virginia Mason Francisan Health Seattle, WA United States; 5 MindMed Inc New York, NY United States; 6 Tufts University School of Medicine Boston, MA United States; 7 Department of Paediatrics University of Toronto Toronto, ON Canada; 8 Department of Pediatric Hematology/Oncology Schneider Children's Medical Center of Israel Petach-Tikva Israel; 9 Department of Pediatrics University of Toronto Toronto, ON Canada; 10 Gasteroentology Unit Oxford University Hospitals NHS Foundation Trust and Biomedical Research Centre Oxford United Kingdom; 11 The Dr. Henry D. Janowitz Division of Gastroenterology Icahn School of Medicine at Mount Sinai New York, NY United States; 12 Department of Neurosurgery Johns Hopkins University School of Medicine Baltimore, MD United States; 13 The Sidney Kimmel Comprehensive Cancer Center at Johns Hopkins Baltimore, MD United States; 14 Princess Margaret Cancer Center University Health Network Toronto, ON Canada; 15 Evidation Health Inc Santa Mateo, CA United States; 16 Sage Bionetworks Seattle, WA United States

**Keywords:** wearables, wearable, mHealth, mobile health, app, apps, application, applications, engagement, adherence, retention, participatory medicine, participatory, DHT, digital health technology, DHTs, digital health technologies, digital health, mobile phone

## Abstract

**Background:**

Wearable digital health technologies and mobile apps (personal digital health technologies [DHTs]) hold great promise for transforming health research and care. However, engagement in personal DHT research is poor.

**Objective:**

The objective of this paper is to describe how participant engagement techniques and different study designs affect participant adherence, retention, and overall engagement in research involving personal DHTs.

**Methods:**

Quantitative and qualitative analysis of engagement factors are reported across 6 unique personal DHT research studies that adopted aspects of a participant-centric design. Study populations included (1) frontline health care workers; (2) a conception, pregnant, and postpartum population; (3) individuals with Crohn disease; (4) individuals with pancreatic cancer; (5) individuals with central nervous system tumors; and (6) families with a Li-Fraumeni syndrome affected member. All included studies involved the use of a study smartphone app that collected both daily and intermittent passive and active tasks, as well as using multiple wearable devices including smartwatches, smart rings, and smart scales. All studies included a variety of participant-centric engagement strategies centered on working with participants as co-designers and regular check-in phone calls to provide support over study participation. Overall retention, probability of staying in the study, and median adherence to study activities are reported.

**Results:**

The median proportion of participants retained in the study across the 6 studies was 77.2% (IQR 72.6%-88%). The probability of staying in the study stayed above 80% for all studies during the first month of study participation and stayed above 50% for the entire active study period across all studies. Median adherence to study activities varied by study population. Severely ill cancer populations and postpartum mothers showed the lowest adherence to personal DHT research tasks, largely the result of physical, mental, and situational barriers. Except for the cancer and postpartum populations, median adherences for the Oura smart ring, Garmin, and Apple smartwatches were over 80% and 90%, respectively. Median adherence to the scheduled check-in calls was high across all but one cohort (50%, IQR 20%-75%: low-engagement cohort). Median adherence to study-related activities in this low-engagement cohort was lower than in all other included studies.

**Conclusions:**

Participant-centric engagement strategies aid in participant retention and maintain good adherence in some populations. Primary barriers to engagement were participant burden (task fatigue and inconvenience), physical, mental, and situational barriers (unable to complete tasks), and low perceived benefit (lack of understanding of the value of personal DHTs). More population-specific tailoring of personal DHT designs is needed so that these new tools can be perceived as personally valuable to the end user.

## Introduction

Wearable digital health technologies (DHTs) [1,2] and mobile apps facilitate the remote, real-world assessment of health including objective signs of disease that are typically confined to health care visits and health care provider interpretation. These specific categories of DHTs, herein referred to as “personal DHTs,” hold promise for transforming health research through the new ability to capture high-resolution, high-frequency, in-the-moment health-related multimodal information in decentralized ways. Through the provision of personal DHTs in clinical care, individuals could be better empowered to navigate their health outside the health care system with greater accessibility, agency, and accuracy than currently possible [1,2]. One of the largest challenges in the future of digital health that involves the use of personal DHTs is end-user engagement. While direct comparisons of engagement in personal DHT research are challenging due to the heterogeneous reporting of retention and adherence factors, and a lack of consensus on a definition of “engagement” [3-6], accumulating evidence supports that so far engagement in the use of personal DHTs has been poor. Specifically, retention in personal DHT research studies and the use of health-related apps is low across diverse populations and applications [7-9]. Further, there is evidence of attrition biases in personal DHT research resulting in insufficient representation of minority populations [7]. In addition to poor retention, personal DHT research studies have low adherence to completing active app-based tasks resulting in large amounts of missing data. This missing data problem results in challenges in artificial intelligence models from insufficient volumes of data to follow individual patterns, and limits app-based context “label” data. This “label” data is crucial for validating passively collected information from personal DHTs, particularly given the early state of the field and as the utility of certain approaches such as knowledge graphs and large language models emerge.

Several personal DHTs health research studies have started to surface [7-12], resulting in the identification of barriers to engagement. These barriers include technical problems with the technology and in collecting the data, usability, privacy concerns, and digital literacy. Many of these barriers point to a need to retain a human element in the research process, and to include an aspect of co-designing with end users. Emerging personal DHT research studies that show better engagement retain some form of “human-in-the-loop” (regular contact with research staff) and co-design or end-user approach [11-15]. Among these studies, retention rates of 80% and higher have been observed, while average adherence to wearable device use and daily app surveys have been shown to be >90% and 70%, respectively [11-15].

The promise of digital health rests on the assumption that end users can be engaged in the long-term use of personal DHTs for health monitoring, yet this remains to be seen among most existing research applications. There have been increasing international calls for the inclusion of patients in the design and conduct of health research [16-18], and this seems particularly relevant for digital health research where the patient is the end user of these new remote tools. In this paper, we report on engagement across 6 unique personal DHT health research studies that adopted different aspects of a participant-centric design, but each with distinct population and design features. The objective is to describe how participant engagement techniques and different personal DHT designs affect participant adherence, retention, and overall engagement in personal DHT health research.

## Methods

### Study Design

In total, 6 personal DHT research studies are included in this quantitative and qualitative analysis of engagement that span diverse populations including a frontline health care population (the stress and recovery in frontline health care workers study) [11]; a conception, pregnancy, and postpartum population (Better Understanding the Metamorphosis of Pregnancy [BUMP] study) [19]; and populations with different diseases including Crohn disease (stress in Crohn: forecasting symptom transitions study), Li-Fraumeni syndrome (stress and LFS: a feasibility study of wearable technologies to detect stress in families with LFS), and patients with pancreatic and central nervous system (CNS) tumors (help enable real-time observations [HERO] in pancreatic [PANC] and CNS tumors studies) [20].

All of these studies were conducted by 4YouandMe—a US-based nonprofit (charitable) organization. 4YouandMe specializes in open-source research into the application of personal DHTs for health and wellness [20]. 4YouandMe has a particular focus on leveraging personal DHTs to empower the patient in navigating their unique disease or life transitional period. These 6 studies were included in this analysis as they reflect all of the completed studies by 4YouandMe at the time of this analysis. Characteristics of these studies can be found in Table 1 and additional methodological detail can be found in Multimedia Appendix 1. All studies involved the use of a bespoke study smartphone app built by 4YouandMe and the use of the Oura smart ring, the Garmin smartwatch, the Apple smartwatch, an Empatica smartwatch, and the Bodyport Cardiac Scale. Details of these devices can be found in Multimedia Appendix 2).

**Table 1:** Characteristics of included studies.

Study and population		Sample size	Age (years), median (IQR)	Active study time (months)	Recruitment	Devices	Average (SD) app daily burden	Compensation	Engagement strategy
**Stress and recovery**									
	Frontline health care workers	365	33.0 (28.0-42.0)	4-6	Remote: Social media and health care organization newsletters	App (iPhone only) Oura ring Garmin watch	5 (1.8) minutes	None (participants completing the study kept the wearable devices)	Biweekly check-in phone calls Investigator-participant Zoom (Zoom Video Communications, Qumu Corporation) calls Participant feedback-driven app changes Coauthorship opportunity
**Stress in Crohn**									
	Patients with Crohn disease	195 (MSSM^a^, N=139; Oxford, N=56)	MSSM (median 29, IQR 24-37), Oxford (median 39, IQR 32-50)	6-9	In-clinic: through inflammatory bowel disease clinics	App (iPhone only) Oura ring Empatica Embrace Bodyport scale	7.7 (1.0) minutes	Yes, participants could keep the ring or receive compensation based on points accumulated	Biweekly check-in phone calls Participant feedback-driven app changes
**HERO-CNS^b^**									
	Patients with CNS^c^ tumors	12	52 (43-56)	7	In-clinic: through cancer specialty clinics	App (iPhone or Android) Oura ring Garmin	5.3 (2.1) minutes	None (participants completing the study kept the wearable devices)	Biweekly check-in (phone or in-clinic)
**HERO-PANC^d^**									
	Patients with pancreatic cancer	26	57 (53-65)^e^	1 to 14 months^f^	In-clinic: through cancer specialty clinics	App (iPhone or Android) Oura ring Garmin Bodyport scale	3.1 (1.9) minutes	None (participants completing the study kept the wearable devices)	Biweekly check-in (phone or in-clinic)
**Stress and LFS^g^**									
	Affected and unaffected family members of a proband with LFS	49	39.0 (7.9-68.0)	6	In-clinic: through cancer specialty clinics	App (iPhone only) Empatica EmbracePlus	2.3 (0.9) minutes	None	Biweekly check-in (phone or in-clinic)
**BUMP^h^**									
	Pregnant individuals (up to 15 weeks)	524	33.0 (30-36)	Up to 12 months	Remote: through patient-provider portals, social media, and community health clinics	App (iPhone or Android) Oura ring Garmin Bodyport scale	5.0 (2.3) minutes	Yes, participants received compensation based on study points accumulated	Biweekly check-in phone calls Investigator-participant Zoom calls Participant feedback-driven app changes
**BUMP-C^i^**									
	Individuals actively attempting to get pregnant	273	34.0 (31-36)	Up to 6 months	Remote: through patient-provider portals, social media, and community health clinics	App (iPhone or Android) Oura ring	3.8 (2.0) minutes	Yes, participants could keep the ring or receive compensation	Biweekly check-in phone calls Investigator-participant Zoom calls Participant feedback-driven app changes

^a^MSSM: Mount Sinai School of Medicine.

^b^HERO-CNS: help enable real-time observations—central nervous system.

^c^CNS: central nervous system.

^d^HERO-PANC: help enable real-time observations—pancreatic cancer.

^e^n=24, 2 unknown.

^f^Until withdrawal, progression, death, or study completion (October 31, 2022).

^g^LFS: Li-Fraumeni syndrome.

^h^BUMP: Better Understanding the Metamorphosis of Pregnancy.

^i^BUMP-C: Better Understanding the Metamorphosis of Pregnancy—Conception.

### Ethical Considerations

All included studies were approved by the local institutional research ethics boards (REB) at their local sites (Multimedia Appendix 1): stress and recovery in frontline health care workers study (institutional review board [IRB], Advarra [4UCOVID1901, Pro00043205]), BUMP study (IRB Advarra Pro00047893), stress in Crohn (Oxford site: Hampshire-A IRAS ID: 269286, Mount Sinai School of Medicine [MSSM] site: IRB of MSSM: GCO 19-1543 | IRB-19-02298), stress and LFS (Sick Kids: REB: 1000072240), HERO-CNS (John Hopkins Medicine IRB IRB00253818), and HERO-PANC (University Hospital Network REB: 20-5211).

### Statistical Analysis

Definitions of adherence in digital health research studies are heterogeneous [3-6]. Consistent criteria for adherence across all included studies were attempted. While many different wearable features could be used as the basis for the use of the device, features that were most reliably monitored were selected. For studies using the Oura smart ring, daily adherence was defined as at least one sleep data event present for the prior night. The Oura ring was only expected to be worn at night for many of the included studies, which is why sleep data were used as the indicator for adherence. For studies using the Garmin smartwatch, daily adherence was defined as step data present for that day. For the Empatica smartwatch, daily adherence was defined as at least one data event (worn properly in a day). Adherence to the Bodyport Cardiac Scale was defined as the proportion of days where a weight event was present divided by the total number of expected follow-up days. Adherence to in-app task completion was defined as the proportion of tasks completed when prompted in the app divided by the total number of tasks that should have been completed over study follow-up. For example, all included studies had a daily survey. In a study with a minimum of 4 months of follow-up expected from participants, the total number of expected daily surveys is approximately 120. For a weekly app survey, the total number of expected surveys for a 4-month study follow-up would be 16. Adherence to biweekly check-in calls was defined as the proportion of calls completed divided by the total number of expected calls over study follow-up. Medians and ranges are described since the adherence distributions were nonnormally distributed. All adherence estimations were performed only among retained participants.

Differences in adherence and retention by sociodemographic characteristics were estimated using *χ*^2^, Fisher exact, Mann-Whitney *U*, and ANOVA tests where appropriate among studies that have sufficient sample sizes (stress and recovery, BUMP, and stress in Crohn). Survival probabilities using the Kaplan-Meier approach were calculated to display the probability of retention over the course of each included study. Retention (total proportion of participants completing the study among all enrolled) is also reported. Additional information on how retention was calculated for each unique study can be found in Multimedia Appendix 3.

## Results

### Description of Included Studies

Study design characteristics of all studies are described in Table 1. All studies included the use of at least one wearable device plus a study app that involved daily, as well as intermittent surveys (daily question prompts, validated questionnaires) and active tasks (cognitive active or physical function tasks [eg, walk tests], video diaries). In all included studies, participants were required to use their own Android or iPhone smartphone for study activities. Recruitment mechanisms differed across studies with some including remote recruitment through digital advertisements on social media, professional organizations and newsletters, and patient portals (stress and recovery, and BUMP), while others recruited patients in-person through specialty clinics (stress in Crohn, HERO studies, and stress and LFS). The daily burden of app active tasks across studies ranged from 2 to 7 minutes. Study follow-up periods across studies ranged from 4 to 18 months. Across all studies except the stress and LFS study, participants were offered to keep some of the study wearable devices (most often the ring and the watch). Further, 2 studies included the option for modest financial compensation (BUMP and stress in Crohn).

All studies included an engagement strategy that centered around a biweekly phone check-in with a consistent engagement specialist that served the purpose of supporting participants, helping them with onboarding, resolving potential technological problems, and discussing and collecting study experience feedback. Additionally, all included studies implemented different strategies that focused on working with participants as co-designers. These strategies included making app changes that were driven by direct participant feedback during active follow-up, offering a “your data” section in the app that allowed participants to track key symptoms over time, hosting optional investigator-participant Zoom calls where participants could meet the study team, receive study updates, preliminary results, and could offer more feedback, and inviting participants to contribute to and be listed as coauthors on published work.

### Adherence by Study Population

Median adherence in engagement phone check-in calls, wearable device use, daily app survey completion, and in-app active tasks can be found in Table 2. Median adherence varied across study populations. The stress in Crohn–MSSM site had a lower adherence on the engagement check-in calls (50%) compared to other studies, many of which had 100% adherence on these calls (Table 2). This study site is herein referred to as the low-engagement cohort. In this low-engagement cohort, median adherence to completing daily app surveys, to wearing the Empatica smartwatch, and to using the Bodyport Cardiac Scale were lower than all other study cohorts that included these studies’ activities (except the BUMP-postpartum cohort). Further, median adherence to using the Oura smart ring was lower in the low-engagement cohort compared to other cohorts except for the postpartum and severely ill cancer populations.

The HERO studies included the most severely ill participants including patients with active diagnoses of CNS and pancreatic tumors. Some HERO participants were undergoing chemotherapy, some had therapy-related complications, some had infections, and some had progressive, life-threatening tumor growth. While the total number of participants in these studies was low, these studies showed low adherence on the daily survey (<55%) and wearable device use (<65% HERO-CNS only). Interestingly, HERO-PANC participants exhibited high wearable device use median adherence (83.3%, IQR 51%-93.2%, Oura and 95.5%, IQR 75.2%-99.2%, Garmin), despite the health status of this population. Further, median adherence to in-app cognitive active tasks was higher among the HERO studies compared to most other studies. Engagement check-in call adherence was also high in the HERO studies. Among the BUMP postpartum cohort, there was consistently lower adherence on all study tasks except for the engagement check-in calls compared to other studies, particularly in comparison to the BUMP prenatal cohort. Specifically, median adherence to the Oura ring, Garmin smartwatch use, and the Bodyport Cardiac Scale in the BUMP-prenatal cohort compared to the BUMP postpartum cohort dropped from 87.2% (IQR 68.7%-96.7%) to 55% (IQR 5.5%-83.7%), 96.7% (IQR 82.9%-100%) to 62.5% (IQR 12.3%-96.4%), and 74.7% (IQR 52%-87.3%) to 33.1% (IQR 8.9%-67.7%), respectively (Table 2).

**Table 2:** Median adherence to study activities across studies.

	Stress and recovery	BUMP-C^a^	BUMP^b^	BUMP-POST^c^	SINC^d^-MSSM^e^	SINC-Oxford	HERO-CNS^f^	HERO-PANC^g^	Stress in LFS^h^
Participants, n	297	98	379	379	117	54	7	19	45
ES^i^ check-ins, median (IQR)	75.0 (57.1-87.5)	100.0 (87.9-100.0)	100.0 (88.4-100.0)	100.0 (100.0-100.0)	50.0 (20.0-75.0)	100.0 (90.9-100.0)	85.7 (78.1-88.2)	100.0 (100.0-100.0)	60.0 (40.0-80.0)
Oura ring, median (IQR)	97.0 (86.0-100.0)	90.6 (76.3-97.7)	87.2 (68.7-96.7)	55.0 (5.5-83.7)	80.5 (37.1-92.4)	98.9 (94.0-99.6)	42.3 (32.0-58.2)	83.3 (51.0-93.2)	—^j^
Garmin watch, median (IQR)	—	—	96.7 (82.9-100.0)	62.4 (12.3-96.4)	—	—	63.3 (54.7-64.3)	95.5 (75.2-99.2)	—
Apple watch, median (IQR)	—	—	98.1 (87.7-100.0)	79.8 (32.4-96.3)	—	—	—	—	—
Empatica watch, median (IQR)	—	—	—	—	26.0 (6.2-64.1)	72.5 (37.1-96.8)	—	—	86.8 (66.7-95.6)
Bodyport scale, median (IQR)	—	—	74.7 (52.0-87.3)	33.1 (8.9-67.7)	38.5 (17.1-64.7)	—	—	79.5 (52.7-88.4)	—
Daily survey, median (IQR)	75.4 (57.2-88.2)	42.4 (24.6-69.7)	60.1 (34.4-81.7)	18.4 (1.0-47.6)	27.9 (10.4-51.9)	70.3 (41.9-84.0)	53.3 (47.8-71.5)	49.1 (20.2-83.4)	62.5 (40.96-82.59)
Reaction rime, median (IQR)	88.9 (75.0-100.0)	43.4 (24.3-72.8)	—	—	30.4 (9.7-50.6)	69.5 (46.6-89.3)	59.0 (50.0-66.7)	62.5 (20.9-86.6)	—
Trail making, median (IQR)	88.9 (71.1-100.0)	46.5 (24.0-73.7)	—	—	28.7 (9.4-50.0)	71.6 (45.0-87.3)	61.5 (52.1-76.5)	38.1 (4.2-76.2)	57.7 (36.8-72.0)
EBT^k^, median (IQR)	—	30.1 (16.2-54.1)	44.6 (22.6-73.9)	6.5 (0.0-33.3)	23.1 (9.1-44.4)	32.1 (0.0-58.6)	—	—	—
N-Back, median (IQR)	—	—	51.4 (24.9-76.4)	8.3 (0.0-44.4)	—	—	—	—	—
Gait task, median (IQR)	—	—	25.0 (0.0-60.0)	0.0 (0.0-0.0)	—	—	24.5 (18.8-62.8)	36.0 (2.2-74.0)	—
Walk test, median (IQR)	—	—	14.3 (0.0-40.0)	0.0 (0.0-0.0)	—	—	23.1 (13.9-60.4)	25.0 (7.8-49.5)	—
Video diary, median (IQR)	—	4.3 (0.0-27.7)	8.3 (0.0-50.0)	0.0 (0.0-0.0)	5.6 (0.0-22.2)	9.4 (0.0-35.1)	25.0 (8.7-77.1)	0.0 (0.0-37.5)	—

^a^BUMP-C: Better Understanding the Metamorphosis of Pregnancy—Conception.

^b^BUMP: Better Understanding the Metamorphosis of Pregnancy.

^c^BUMP-POST: Better Understanding the Metamorphosis of Pregnancy—Postpartum.

^d^SINC: stress in Crohn.

^e^MSSM: Mount Sinai School of Medicine.

^f^HERO-CNS: help enable real-time observations—central nervous system.

^g^HERO-PANC: help enable real-time observations—pancreatic cancer.

^h^LFS: Li-Fraumeni syndrome.

^i^ES: engagement specialist.

^j^Not available.

^k^EBT: emotional bias test.

### Adherence by Study Activity

There were differences in adherence rates across different study activities. Adherence to wearable device use was consistently higher across studies compared to in-app activities, which is not surprising given the passive nature of these devices. Excluding the postpartum and HERO-CNS study, median adherence to Oura ring use was >80% across all studies, and as high as 99% (IQR 94.9%-99.6%; stress in Crohn-Oxford site; Table 2). There were also differences in adherence across specific wearable devices. Garmin and Apple smartwatch adherence was >95% in BUMP pregnant individuals and HERO-PANC participants, while median adherence for the Empatica Watch was lower among the studies that used this device (stress in Crohn-Oxford, 72.5%, IQR 37.1%-96.8%; stress in Crohn-MSSM, low-engagement cohort, 26%, IQR 6.2%-64.1%; and stress in LFS, 86.8%, IQR 0.7%-0.9%). Median adherence to the Bodyport Cardiac Scale was 74.7% (IQR 52%-87.3%) among BUMP pregnant individuals and 79.5% (IQR 52.7%-88.4%) in HERO-PANC participants (Table 2). Excluding the postpartum and HERO study populations and the low-engagement cohort, in-app daily survey adherence was >60% for all studies (Table 2). Finally, adherence to in-app active tasks was lower in general compared to other activities such as wearable device use or in-app surveys. Tasks that involved walking (gait and walk task) or speaking (video diaries) showed lower adherence compared to other active tasks (eg, cognitive and emotional bias tasks; Table 2).

### Adherence by Study Recruitment and Engagement Strategy

There did not appear to be any meaningful difference in median adherence rates across study activities by study recruitment methods (in-clinic vs remote) or follow-up time. Further, 2 studies that included modest financial compensation in addition to engagement strategies showed higher adherence rates compared to some of the other studies (ie, BUMP and stress in Crohn), but the impact of compensation is difficult to disentangle from other study characteristics such as population differences, and these studies did not show superior adherence rates compared to the stress and recovery study that did not offer financial compensation.

### Retention

The median proportion of participants retained in the study across the 6 studies was 77.2% (IQR 72.6%-88%; Table 3). The probability of staying in the study stayed above 80% for all studies during the first month of study participation and stayed above 50% for the entire active study period across all studies (Multimedia Appendix 4).

**Table 3:** Retention across studies.

Study	Proportion retained at study completion, retained/enrolled (%)
Stress and recovery	297/365 (81.4)
BUMP-C^a^	134/187 (72.7)^b^
BUMP^c^	379/524 (72.3)
Stress in Crohn-MSSM^d^	117/139 (84.2)
Stress in Crohn-Oxford	54/56 (96.4)
HERO-CNS^e^	7/12 (58.3)
HERO-PANC^f^	19/26 (73.1)^g^
Stress and LFS^h^	45/49 (91.8)

^a^BUMP-C: Better Understanding the Metamorphosis of Pregnancy—Conception.

^b^Only includes participants who were enrolled in the Better Understanding the Metamorphosis of Pregnancy—Conception-specific app.

^c^BUMP: Better Understanding the Metamorphosis of Pregnancy.

^d^MSSM: Mount Sinai School of Medicine.

^e^HERO-CNS: help enable real-time observations—central nervous system.

^f^HERO-PANC: help enable real-time observations—pancreatic cancer.

^g^Help enable real-time observations—pancreatic cancer has unique factors to consider when interpreting the proportion retained until study completion, since the study aimed to monitor patients until they developed progressive disease or died, or the study end date (October 31, 2022; see Multimedia Appendix 3).

^h^LFS: Li-Fraumeni syndrome.

### Adherence and Retention by Participant Sociodemographic Characteristics

Median adherence for the Oura smart ring, a smartwatch (Garmin, Apple, and Empatica), and the Bodyport Cardiac Scale was lower among younger participants compared to older participants across most studies (Multimedia Appendix 5). Specifically, Oura smart ring adherence was significantly lower in those aged 18-25 years compared to those aged ≥26 years in the BUMP study (*P*=.03) and stress in Crohn-MSSM studies (*P*=.02), and was lower in the BUMP-C and stress and recover studies, but this difference was not statistically significant at *P*=.59 and *P*=.08, respectively. Median adherence for Apple smartwatch use was significantly lower in those aged 18-25 years compared to those aged ≥26 years in the BUMP study (*P*=.02), while median adherence for Garmin smartwatch use was lower but not statistically significant (*P*=.06). Median adherence for the Bodyport Cardiac Scale was significantly lower in those aged 18-25 years compared to those aged ≥26 years in BUMP (*P*<.005) and stress in Crohn-MSSM (*P*<.006).

In the BUMP study, Black or African American ethnicity had significantly higher median adherence to completing the in-app daily survey compared to other race or ethnicity groups (*P*=.01). This trend was observed in the stress and recovery study (*P*=.07) and the stress in Crohn-MSSM study (*P*=.24), although the difference was not statistically significant. In contrast, median adherence to Oura smart ring, smartwatch, and Bodyport Cardiac Scale use was lower among Black or African American individuals compared to other race or ethnicity groups, although these differences were not statistically significant (Multimedia Appendix 5).

Retention did not significantly differ by age group or gender (Multimedia Appendix 6).

Retention likelihood was significantly different by race or ethnicity groups in BUMP-C (*P*<.001) and BUMP (*P=*.001). Specifically, participants of White ethnicity were more likely to stay in the study in both BUMP-C and BUMP, while participants reporting their race or ethnicity as either unknown or not reporting this item were less likely to be retained (Multimedia Appendix 6).

### Barriers to Engagement (Qualitative Synthesis of Participant Feedback)

**Figure 1:**
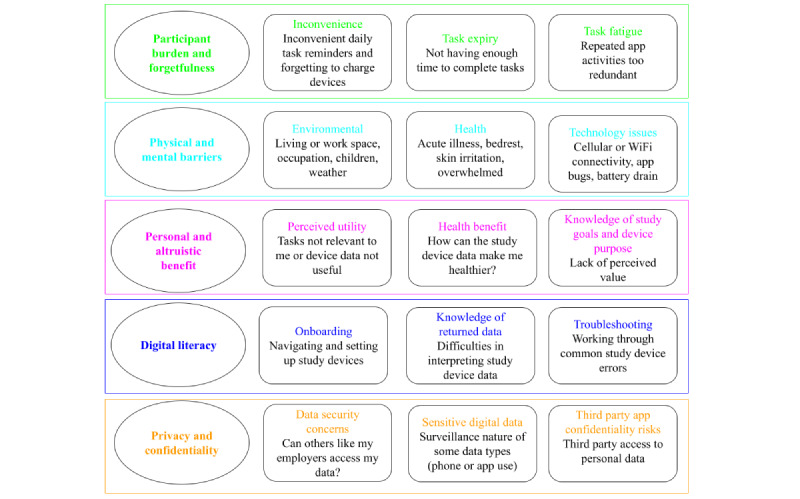
Five key participant engagement barrier themes.

Figure 1 describes key themes that impacted participant retention, adherence, and overall engagement that cut across all included studies. These themes include participant burden and forgetfulness, digital literacy, physical and mental barriers, personal and altruistic benefits, and privacy and confidentiality. Qualitative feedback from participants, research staff, and investigators across these 5 themes is summarized in Multimedia Appendix 7. The top three barriers to engagement in active study tasks were (1) participant burden and in particular fatigue with the repetitiveness of tasks; (2) physical or mental and situational barriers that prevented the ability to complete tasks; and (3) personal and altruistic benefit, namely the perception that the use of the personal DHTs was not personally useful for a health benefit or a lack of understanding as to why and how certain features (eg, heart rate variability) could be useful to track for health benefit. Qualitative feedback from participants in the 2 cohorts demonstrating lower adherence (HERO-PANC and BUMP post partum) suggested that while participants were highly engaged, they were either too ill, distracted, or tired to complete many of the study activities while navigating a serious illness or the early postpartum period.

## Discussion

### Principal Findings

Evidence across 6 unique and diverse studies involving the longitudinal use of personal DHTs supports that participant-centric engagement strategies aid in participant retention and maintaining good adherence in some populations. These strategies centered around (1) human contact with an engagement specialist as often as every 2 weeks, (2) investigator-participant meetings during active study follow-up, (3) offering returned symptom data in the app, (4) inviting participants to contribute as coauthors in published work, and (5) real-time modifications to the study app based on participant feedback.

In the majority of included studies, the probability of staying in the study stayed above 90% for the first month and stayed above 50% for active study periods for all studies. Lower retention or adherence was observed among studies that included a severely ill cancer population and a postpartum population. Barriers to participation in these cohorts were largely the result of physical and situational roadblocks. Excluding studies of a severely ill and postpartum population and the low-engagement cohort in the stress in Crohn study, adherence to Oura smart ring and Garmin smartwatch use was 80% and as high as 99% in some cohorts, while adherence to the Bodyport Cardiac Scale was 75% in a pregnant population. This supports that different populations can successfully be engaged in the use of active app assessments and wearable devices in the long term with adequate support.

Retention and adherence rates observed in these studies are higher than typically reported by other personal DHT research studies [7-9,12,13,21]. For example, a review of 8 large app-based DHT research studies in the United States reported that the probability of staying in the study dropped to or below 50% after the first 4 weeks of participation for all included studies [7]. Further, across the 8 included studies in this review, >50% of participants did not engage with the app for at least 7 days. Another large app-based study in the United States, the Warfighter Analytics Using Smartphones for Health study that collected daily active and passive app data reported a median retention of 45.2% (38/84 days), while the probability of staying in the study hit 50% at approximately 5.5 weeks [10]. A large app-based study in the United Kingdom (cloudy with a chance of pain study) involving daily active app assessments reported that 64% of participants fell into the low engagement or no engagement categories after baseline [12]. The RADAR study [14], a multinational study involving active and passive assessments from an app, and a Fitbit reported comparable retention results among participants with major depression to those reported here. This study reported a retention rate of 54.6% for 43 weeks of study participation; however, the probability of staying in the study stayed above 75% for the first several months of participation (~6 months). While the active app assessments in this study only included assessments every 2 weeks as opposed to daily assessments, this study additionally included aspects of a participant-centric design, which may have contributed to the higher reported retention [15].

Taken together, in comparison to other published personal DHT research studies, the 6 studies included in this paper reflect higher levels of engagement. Importantly, the included studies in this analysis involved high burden designs in comparison to other studies that request, for example, weekly or biweekly active tasks of participants [14] or only involve the use of a smartwatch. Specifically, across the included studies here, participants were expected to complete on average 4.6 (SD 1.62) minutes a day of app activities in addition to continuously using multiple wearable devices.

While different variations of participant-centric strategies were used across the 6 included studies, a key common feature was a biweekly check-in call with an engagement specialist. These calls served the purpose of providing support and building rapport with participants, working through onboarding and technological issues with study devices, tracking adherence, and receiving study-related feedback from participants. Numerous challenges arise in the conduct of remote, personal DHT research, and without frequent check-in and semiregular data monitoring by research staff, knowledge of these issues is a black box. The most significant drop in retention in personal DHT research studies tends to be during the first few weeks of participation [7]. These early onboarding weeks are crucial in working with participants to ensure they can get into a rhythm of participation. The passive sensing nature of personal DHTs has much potential to inform new objective measures of health, however, are not always intuitively understood as personally important for unique diseases (eg, heart rate variability or phone screen time). Personal DHT studies allow for “light touch” research approaches that enable data collection without traditional research coordinator contact, but this may come with a cost that inadvertently creates a less engaging study environment for participants and limits the opportunity to help participants understand the value in their participation. Of the included 6 studies, 1 cohort had much lower engagement on the check-in calls (50% adherence) compared to other included studies and, in turn, consistently demonstrated lower adherence to study-related activities. Still, even with extensive engagement designs, populations that had physical, mental, and situational barriers to study task completion (ie, severely ill, postpartum mothers) showed lower adherence to wearable device use and active smartphone tasks compared to other study populations. Top reported barriers to engagement included participant burden, physical, mental, and situational barriers, and low perceived value of personal DHTs for health care. These engagement barriers have been reported in previous literature [8,9] relating to DHT research and in the use of DHT interventions. However, the conveyed importance of the perceived value of the approach among participants in the current analysis is noteworthy. Given the foreign nature of personal DHTs for many individuals, particularly older populations, further work is needed to co-design and educate end users on the potential value of self-monitoring unique health-related data.

Irrespective of the engagement approach, adherence to in-app surveys and tasks was lower than wearable device use, which is not surprising given the higher burden related to in-app activities. The self-reported information captured from frequent or momentary in-app assessments is extremely valuable as context information. This context information or “label” data is useful for validating objectively captured information, yet remains the most difficult to capture in sufficient detail. Further, certain in-app activity adherences were consistently lower than others. Namely, activities that required the user to be active (walk in a straight line or complete a video diary) were low across studies. Still, adherence to daily in-app surveys was >60% for all studies excluding the postpartum and HERO study populations.

### Limitations

This quantitative and qualitative analysis compared observational data across different digital health studies. However, no true comparison cohort that did not include engagement strategies was included. Therefore, the inferred casualty of participant check-ins with engagement specialists on retention and adherence rates cannot be not concluded. We are formally testing whether the biweekly check-in significantly increases adherence and retention in an ongoing study with an appropriate comparison arm without check-in support (NCT05753605). One of the included studies (stress and recovery) was conducted during the early 2020 COVID-19 pandemic. There is some evidence that engagement in research was higher during the early pandemic time periods [22]. It cannot be ruled out that the higher observed retention and adherence in this study compared to others was not due to this potential time period bias. The stress in the Crohn-Oxford site included a population of patients some of whom were already engaged in the use of web-based monitoring of symptoms. In turn, this could have contributed to the high retention and higher adherence observed at this site compared to the other stress in the Crohn-MSSM site. The results presented on barriers to engagement were primarily qualitative and collected from conversations with participants, research staff, and investigators across studies.

### Conclusions

Globally, mobile apps are used for a variety of purposes in everyday life, while the use of smartwatches for activity monitoring is gaining increasing popularity. However, the use of these tools for health remains a challenge. These findings support that human support via phone and other participant-centric engagement strategies centered on giving back to participants and working with them as co-designers can support sufficient retention and adherence in personal DHT research across diverse populations. This has implications for the utility and potential necessity of a digital support worker in digital health care, as highlighted by others [23]. A power of personal DHTs is enabling the patient to be in control of their health through self-monitoring, but this new role comes with a responsibility. This important shift in role from doctor to patient outlines how crucial it is to include patients in the early design phase of personal DHT health research. Further work is needed to inform app designs that support habitual forming activities around task completion so that app-related activities become a part of participants’ daily routine and are perceived as personally valuable.

## Appendix

Multimedia Appendix 1 Study descriptions.

Multimedia Appendix 2 Study wearable devices.

Multimedia Appendix 3 Retention calculations.

Multimedia Appendix 4 Probability of retaining in the study across studies.

Multimedia Appendix 5 Median adherence to study activities stratified by sociodemographic characteristics.

Multimedia Appendix 6 Sociodemographic differences in participants who were retained versus not retained.

Multimedia Appendix 7 Qualitative feedback from participants, research staff, and investigators surrounding barriers to engagement in digital health research, summarized across 6 unique studies.

## Abbreviations

BUMP: Better Understanding the Metamorphosis of Pregnancy
CNS: central nervous system
DHT: digital health technology
HERO: help enable real-time observation
IRB: institutional review board
LFS: Li-Fraumeni syndrome
MSSM: Mount Sinai School of Medicine
PANC: pancreatic cancer
REB: research ethics board
