# Supplementary material for: Value of Engagement in Digital Health Technology Research: Evidence Across 6 Unique Cohort Studies
Source: J Med Internet Res. 2024 Sep 3;26:e57827. doi: 10.2196/57827 (PMC11408887; doi:10.2196/57827)
Supplement: Multimedia Appendix 1 [file jmir_v26i1e57827_app1.docx]

**Multimedia appendix 1**

**Stress and Recovery in Frontline Healthcare Workers Study**

**Goal/Objective:** The goal of the Stress and Recovery study was to determine the feasibility of tracking “digital stress responses” to benefit frontline workers in the future in the development of tools that might offer early warning signs of risky health states.

**Description of the study:** The Stress and Recovery study is a Bill and Melinda Gates Foundation funded feasibility cohort study aimed at informing better understanding of individual stress responses and recovery from stress in frontline healthcare workers. This study launched in May, 2020 and was completed in Nov, 2020. 365 frontline healthcare workers were recruited digitally through a variety of social media outlets and email notifications through medical based organizations such as the Mayo Clinic and the American Nurses Association. This study used the Oura smart ring, (worn off-shift) to collect semi-continuous passive and intermittent active biometric measurements alongside daily to monthly surveys and active tasks completed from a study smartphone app for 4-6 months. Some participants completed sub-arms involving a choice of a 4-week lifestyle intervention (exercise vs meditation), providing a hair sample for cortisol analysis, and the continuous wear of a Garmin smartwatch (worn on shift) for 4-weeks. Participants were supported by bi-weekly engagement phone calls by research staff, were invited to participate in an investigator - participant anonymous zoom meeting, and were all invited to co-author a scientific article related to the study.

**Ethical approval:** Institutional Review Board, Advarra (4UCOVID1901, Pro00043205)

**ClinicalTrials.Gov**: [NCT04713111](https://clinicaltrials.gov/ct2/show/NCT04713111)

**Better Understanding the Metamorphosis of Pregnancy (BUMP)/ BUMP-Conception(C))**

**Goal/Objective:** The BUMP study was designed to derive novel insights and deeper understanding of the pre-pregnancy and pregnancy experience with an aim to follow eight core symptoms (emesis, fatigue, edema, mood, cognition, dyspnea, gait, and blood pressure changes) through the use of connected devices such as wearable devices, smart home scales and smartphone apps.

**Description of the study:** This BUMP study included a BUMP cohort recruiting women up to and including 15 weeks pregnant, and a BUMP-Conception (C) cohort recruiting women who are trying to become pregnant. Participants were followed up to 3 months postpartum. Participants were recruited through a variety of different channels in the United States including a patient provider platform, women attending Community Health Centers, and through social media campaigns. In both cohort’s passive and active measures of physiological, psychological, cognitive, sleep and physical symptoms were collected. Objective and subjective measures of health were collected from a central study app, an Oura smart ring, a Garmin or Apple smart wristwatch (BUMP cohort only), and the Bodyport Cardiac Scale (BUMP cohort only). These digital data were complimented by information derived from standard of care clinical visits, while participants were provided with knowledge (insights about their own symptoms), and support by phone check-ins with engagement specialists during participation. Participants were also invited to participate in an investigator - participant anonymous zoom meeting.

**Ethical approval:** Institutional Review Board, Advarra (Pro00047893)

**Clinicaltrials.gov:** NCT04714905

**Stress in Crohn’s: Forecasting Symptom Transitions**

**Goal/Objective:** Day to day acute changes, as well as the longer-term progression of Crohn’s disease are known to be somehow affected by stress, but the details of this relationship is poorly understood. The Stress in Crohn’s study was designed to explore the feasibility of following stress using wearable devices to predict the risk of upcoming symptom changes in Crohn’s disease.

**Description of the study:** The Stress in Crohn’s study is a Helmsley Trust funded multi-site longitudinal study that launched in February, 2021. 171 patients with clinician confirmed Crohn’s disease were followed for 6-9 months between two gastroenterology clinical sites: the John Radcliffe Hospital in Oxford, UK, and the Mount Sinai School of Medicine. Participants were recruited in person during in-clinic visits. This study included continuous physiological and behavioral measurements using a study companion app installed on the participant's own phone (Android or iPhone), which tracked both passive sensor measures, active surveys and cognitive active tasks. Additionally, an Empatica Smartwatch, the Oura smart ring, and the Bodyport Cardiac Scale (MSSM site only) were provisioned to participants to use continuously during study active time. Clinical data from the patient's electronic medical charts were extracted on Crohn’s disease symptom progression. Participants were supported through bi-weekly phone calls by research staff.

**Ethical Approval:** Oxford site: Hampshire-A IRAS ID: 269286, Mount Sinai School of Medicine site: IRB of MSSM: GCO 19-1543 | IRB-19-02298

**Stress and Li Fraumeni Syndrome (LFS) Study**

**Goal/Objective:** Li-Fraumeni Syndrome (LFS) is associated with considerable mortality and morbidity with high probabilities of developing cancer at an early age. Stress in families with LFS is an almost definite occurrence, with personal and family member cancer anticipation, screening fatigue, anxiety about false negative and false positive findings, the caregiving for affected family members, and the high morbidity related to cancer itself. The aim of this study was to determine the feasibility of using digital tools to detect and track stress in families with Li-Fraumeni Syndrome.

**Description of the study:** 45 participants including patients with a diagnosis of LFS and their affected and unaffected family members were enrolled over a 6-month period into a 6-month follow-up study. Patients were recruited in person from cancer clinics at the Princess Margaret Cancer Centre and The Hospital for Sick Children in Toronto, Canada. This study included a REDCap smartphone app with daily and intermittent surveys and active tasks alongside the Empatica EmbracePlus smartwatch. Participants were supported through bi-weekly to monthly phone calls by an on-site clinician.

**Ethical Approval:** Sick Kids: Research Ethics Board: #1000072240

**Help Enable Real Time Observations - Central Nervous System Tumors (HERO-CNS)**

**Goal/Objective:** Patients with high grade gliomas endure devastating disease processes with high symptomatic burden and frequency of progression and recurrence. The aim of this study was to assess the feasibility to detect and track symptom transitions preceding tumor recurrences using connected digital tools to develop a sensor map of symptom progression.

**Description of the study:** The HERO-CNS study was a Mark Foundation funded prospective cohort feasibility study. 12 patients with high grade gliomas were recruited from John Hopkins Medical Center in Baltimore, US and were followed for up to seven months. Participants were recruited in person during in-clinic visits. Semi-continuous passive and active measurements (when possible before surgery/radiation therapy/chemotherapy) using a bespoke 4YouandMe smartphone app to collect and transmit data downloaded onto the participants personal mobile phone, alongside the Oura smart ring and a Garmin smartwatch were collected. The app enabled participants to monitor key symptoms during study active time. Bi-weekly to monthly support phone calls by on-site research coordinators were conducted.

**Ethical Approval:** John Hopkins Medicine Institutional Review Board (# IRB00253818)

**Help Enable Real Time Observations - Pancreatic Cancer (HERO-Panc)**

**Goal/Objective:** Pancreatic cancer is challenging because of the high symptom burden and treatment-related toxicities. The primary objective of the HERO-Panc study was to assess the feasibility of a digital approach to assess inter- and intra-patient variability in biometric measurements before tumor progression is detected by more classical approaches.

**Description of the study:** The HERO-Panc study is a prospective cohort feasibility study. 26 patients with a diagnosis of pancreatic cancer who intended to start chemotherapy treatment were enrolled from the McCain Centre for Pancreatic Cancer at the Princess Margaret Cancer Centre in Toronto, Canada. This study utilized a 4YouandMe study app downloaded onto participants' personal mobile phones alongside the Oura smart ring, the Garmin smartwatch and the Bodyport Cardiac Scale. The app enabled participants to monitor key symptoms during study active time. Bi-weekly to monthly support phone calls by on-site research coordinators were conducted.

**Ethical Approval:** University Hospital Network (UHN) Research Ethics Board: #20-5211
