# Supplementary material for: Value of Engagement in Digital Health Technology Research: Evidence Across 6 Unique Cohort Studies
Source: J Med Internet Res. 2024 Sep 3;26:e57827. doi: 10.2196/57827 (PMC11408887; doi:10.2196/57827)
Supplement: Multimedia Appendix 2 [file jmir_v26i1e57827_app2.docx]

**Multimedia appendix 2**

**Oura Smart ring**

The Oura Ring (<https://ouraring.com/>) is a lightweight, durable titanium device worn on the finger that includes a temperature sensor, a gyroscope, a 3D accelerometer and an infrared optical pulse sensor. The device collects a variety of physiological, activity and sleep features. For this analysis the following features were examined: average night time heart rate, average night time heart rate variability calculated from the rMSSD method, average night time respiration rate, night time body temperature deviation (average temperature relative to baseline normal range), and sleep efficiency (proportion of sleep period spent asleep).

**Garmin Smart watch**

The Garmin Venu Sq Smartwatch ((<https://www.garmin.com/>) includes a 3-axis accelerometer, 3-axis gyroscope, Optical heart rate monitor, Altimeter and a Vibration motor. The device is made of a lightweight material similar to that used in many sports watches and is able to measure physiologic and activity features. For this analysis, the following features were examined: daily heart rate, respiration rate and steps.

**Empatica EMBRACE**

The Empatica EMBRACE (http://www.empatica.com) is a smart wristband made of a flexible, durable material similar to that used in many sports watches, and includes a watch case and buckle. The device syncs automatically and wirelessly to most iOS and Android devices using Bluetooth LE wireless technology. In addition to collecting common physiological data such as heart rate, respiration rate and temperature, the Empatica EMBRACE collects electrodermal activity (skin conductance).

**Bodyport Smart scale**

The Bodyport cardiac scale (<https://bodyport.com/>) comprises a physical platform on which the user stands with bare feet. Four electrodes located on the top surface of the platform are used to obtain three biological signals from the user’s body. The first is a passive electrical signal and is similar in origin to an electrocardiograph. The second reflects pulsatile blood flow and is determined by measuring small changes in the electrical resistance through the legs. The third signal measures blood flow velocity through the Aorta and reflects mechanical function of the heart. From these signals, the scale measures several cardiovascular and metabolic markers. For this analysis, we examined weight, body peripheral fluid levels and bodyport sway velocity.
