# Supplementary material for: Value of Engagement in Digital Health Technology Research: Evidence Across 6 Unique Cohort Studies
Source: J Med Internet Res. 2024 Sep 3;26:e57827. doi: 10.2196/57827 (PMC11408887; doi:10.2196/57827)
Supplement: Multimedia Appendix 3 [file jmir_v26i1e57827_app3.docx]

**Multimedia appendix 3**

**Stress and Recovery in Frontline Healthcare Workers**

| **Total Screened** | 964 |  |
| --- | --- | --- |
| **Total Eligible** | 729 | (729/964) 75.62% |
| **Total Excluded** | 235 | (235/964) 24.38% |
| Android User | 121 | 51.49% |
| No Direct Patient Care | 26 | 11.06% |
| Work Not Impacted | 26 | 11.06% |
| Known Prior COVID-19 | 25 | 14.89% |
| Time Commitment | 21 | 8.94% |
| **Other☆** | 16 | 6.81% |
| **Total Enrolled** | 383 | (383/729) 52.54% |
| **Enrolled (Study Sample)** | 365 | (365/383) 95.30% |
| **Total Retained◇** | 297 | (297/365) 81.37% |
| **Sample Attrition** | 68 | (68/365) 18.63% |
| Withdrew* | 8 | 2.19% / 11.76% |
| Dropped Out** | 25 | 6.85% / 36.76% |
| LTFU*** | 35 | 9.59% / 51.47% |
| **Study Period (months)** | (Med [Min,Max]) |  |
| **Overall (N=365)** | 3.80 [0.21, 6.00] |  |
| **Retained** (N=297) | 4.20 [2.50, 6.00] |  |
| **Attrition** (N=68) | 2.05 [0.21, 3.57] |  |
| ☆ e.g., unable to use device(s), limited English proficiency  ◇Participation ≥ 4M and/or End Date = 12/1/2020  *Study team ended participation due to change in eligibility (e.g., no direct patient care, furloughed, loss of ring)  **Participant asked to end their participation (e.g., doesn't like ring, time commitment, no longer interested)  ***Stress & Recovery-specific LTFU criteria and protocol | | |

LTFU: loss to follow-up; Med: median

**Better Understanding the Metamorphosis of Pregnancy (BUMP)**

| **Total Screened** | 1115 |  |
| --- | --- | --- |
| **Total Eligible** | 570 | 51.12% |
| **Total Excluded** | 545 | 48.88% |
| 16+ Weeks Pregnant | 207 | 37.98% |
| Unable to Reach | 207 | 37.98% |
| Not Currently Pregnant | 43 | 7.89% |
| Unable to Use Devices | 13 | 2.39% |
| **Other☆** | 75 | 13.76% |
| **Total Enrolled** | 524 | 91.93% |
| **Total Retained◇** | 379 | 72.33% |
| **Total Attrition** | 145 | 27.67% |
| Withdrew* | 39 | 26.90% |
| Dropped Out | 43 | 29.66% |
| Participation Through Birth** | 50 | 34.48% |
| LTFU*** | 13 | 8.97% |
| **Study Period (months)** | (Med [Min,Max]) |  |
| **All Study Sample (N=524)** | 9.0 [0.0 22.1] |  |
| **Retained** (N=379) | 9.5 [5.9, 22.1] |  |
| **Attrition** (N=145) | 4.3 [0.0, 15.0] |  |
| ☆ e.g., time commitment, compensation amount, EMR privacy concerns, non-US resident, etc.  ◇Participation ≥ 1 month postpartum  *Study team ended participation due to change in eligibility (e.g., no longer pregnant)  ** Participation through birth with <4w participation postpartum  ***BUMP-specific LTFU criteria and protocol | | |

BUMP: Better Understanding the Metamorphosis of Pregnancy; EMR: electronic medical record; LTFU: loss to follow-up; Med: median

**Better Understanding the Metamorphosis of Pregnancy-Conception (BUMP-C)**

| **Total Screened** | 541 |  |
| --- | --- | --- |
| **Total Eligible** | 282 | 52.13% |
| **Total Excluded** | 259 | 47.87% |
| Age > 40y | 204 | 78.16% |
| Not Currently TTC | 24 | 9.20% |
| Non-US Resident | 17 | 6.51% |
| **Other☆** | 16 | 6.13% |
| **Total Enrolled in BUMP-C** | 273 | 95.45% |
| **Enrolled in BUMP-C Appº** | 187 | 68.50% |
| **Total Retained** | 134 | 71.66% |
| **Total Attrition** | 53 | 28.34% |
| Withdrew* | 14 | 26.42% |
| Dropped Out | 39 | 73.58% |
| **Study Period (months)** | (Med [Min,Max]) |  |
| **Enrolled in BUMP-C App (N=187)** | 4.3 [0.0, 11.9] |  |
| **Retained** Sample (N=98) | 6.2 [4.0, 11.9] |  |
| **Attrition** (N=53) | 0.9 [0.0, 3.3] |  |
| ☆ e.g., time commitment, compensation amount, privacy concerns, LEP  ºParticipants enrolled before 8/21/22 (n=86) joined the BUMP app vs. the BUMP-C app  ◇Participation ≥ 4 months in BUMP-C in the BUMP-C App  *Study team ended participation due to change in eligibility (e.g., no longer actively TCC) | | |

BUMP-C: Better Understanding the Metamorphosis of Pregnancy-Conception; LEP: limited english proficiency; Med: median; TTC: trying to conceive

**Stress in Crohn’s: Forecasting Symptom Transitions**

Mount Sinai School of Medicine Site

| **Total Screened** | 162 |  |
| --- | --- | --- |
| **Total Eligible** | 159 | 98.15% |
| **Total Excluded** | 3 | 1.85% |
| Unable to Wear Device(s) | 2 | 66.67% |
| Currently Trying to Conceive | 1 | 33.33% |
| **Total Enrolled** | 139 | 87.42% |
| **Total Retained** | 117 | 84.17% |
| **Total Attrition** | 22 | 15.83% |
| Dropped Out | 14 | 10.07% / 63.64% |
| LTFU*** | 8 | 5.76% / 36.36% |
| **Study Period (months)** | (Med [Min,Max]) |  |
| **Enrolled in SinC (MSSM) (N=139)** | 9.2 [1.8, 23.5] |  |
| **Retained** (N=117) | 9.6 [1.9, 23.5] |  |
| **Attrition** (N=22) | 6.0 [1.8, 14.2] |  |
| ***SINC-MSSM-specific LTFU criteria and protocol | | |

LTFU: loss to follow-up; Med: median; SINC-MSSM: Stress in Crohn’s-Mount Sinai School of Medicine

John Radcliffe, Oxford site

| **Total Screened** | 56 |  |
| --- | --- | --- |
| **Total Eligible** | 56 |  |
| **Total Excluded** | 0 |  |
| **Total Enrolled** | 56 | 100.00% |
| **Total Retained** | 54 | 96.43% |
| **Total Attrition** | 2 | 3.57% |
| Dropped Out | 2 |  |
| **Study Period (months)** | (Med [Min,Max]) |  |
| **Enrolled in SinC (Oxford) (N=56)** | 5.6 [0.2, 9.0] |  |
| **Retained** (N=54) | 5.8 [2.3, 9.0] |  |
| **Dropped Out** (N=2) | 1.1 [0.2, 2.0] |  |

LTFU: loss to follow-up; Med: median; SinC: Stress in Crohn’s

**Help Enable Real Time Observations - Central Nervous System Tumors (HERO-CNS)**

| **Total Screened** | 31 |  |
| --- | --- | --- |
| **Total Eligible** | 12 |  |
| **Total Excluded** | 19 |  |
| **Total Enrolled** | 12 |  |
| **Total Retained** | 7 | 58.33% |
| **Total Attrition** | 5 | 41.67% |
| Dropped Out | 3 | 60.00% |
| LTFU | 1 | 20.00% |
| Death | 1 | 20.00% |
| **Study Period (months)** | (Med [Min,Max]) |  |
| **Enrolled in HERO-CNS (N=12)** | 4.9 [2.1, 12.0] |  |
| **Retained** (N=7) | 6.1 [4.1, 12.0] |  |
| **Attrition** (N=5) | 4.1 [2.1, 7.0] |  |

HERO-CNS: Help Enable Real Time Observations - Central Nervous System Tumors; LTFU: loss to follow-up; Med: median

**Help Enable Real Time Observations - Pancreatic Cancer (HERO-PANC)**

| **Total Screened** | 293 |  |
| --- | --- | --- |
| **Total Eligible** | 36 | 12.29% |
| **Total Excluded** | 257 | 87.71% |
| **Total Enrolled** | 26 | 72.22% |
| **Total Retained** | 19 | 73.10% |
| **Total Attrition** | 7 | 26.92% |
| Withdrew consent | 4 | 57.14% |
| Stopped participation | 3 | 42.86% |
| **Study Period (months)** | (Med [Min,Max]) |  |
| **Enrolled in HERO-PANC** (N=26) | 7.2 [1.0, 13.1] |  |
| **Retained** (N=19) | 7.1 [1.5, 14.4] |  |
| **Attrition** (N=7) | 8.4 [1.0, 13.1] |  |
| *During study period before completing participation; note: a number of other deaths were noted but occurred in month(s) after completion of study | | |

HERO-PANC: Help Enable Real Time Observations - Pancreatic cancer; Med: median

**Stress and LFS Study**

| **Total Screened** | 82* |  |
| --- | --- | --- |
| **Total Eligible** | 59 | 71.95% |
| **Total Excluded** | 23 | 28.05% |
| Too Stressful / Daily LFS Reminder | 7 | 30.43% |
| Declined** | 6 | 26.09% |
| New / Recent Cancer Diagnosis | 3 | 13.04% |
| Non-Compatible Phone | 3 | 13.04% |
| No Response After Initial Contact | 3 | 13.04% |
| Relocation | 1 | 4.35% |
| **Total Enrolled***** | 49 | 83.05% |
| **Total Retained** | 45 | 91.84% |
| **Total Attrition** | 4 | 8.16% |
| Dropped Out | 4 |  |
| **Study Period (months)** | (Med [Min,Max]) |  |
| **Enrolled in Stress & LFS (N=49)** |  |  |
| **Retained** (N=45) |  |  |
| **Attrition** (N=4) |  |  |
| *22 Families with 67 Individuals and 15 Individuals  **Did Not Specify (4) + New Child Cancer Diagnosis (1) + Second Smartwatch (1)  ***10 Eligible Did Not Enroll & Start Study (watch/phone pairing issues (6) + withdrew consent (4)) | | |

LFS: Li Fraumeni Syndrome; Med: median
