# Supplementary figures and images for: Value of Engagement in Digital Health Technology Research: Evidence Across 6 Unique Cohort Studies
Source: J Med Internet Res. 2024 Sep 3;26:e57827. doi: 10.2196/57827 (PMC11408887; doi:10.2196/57827)

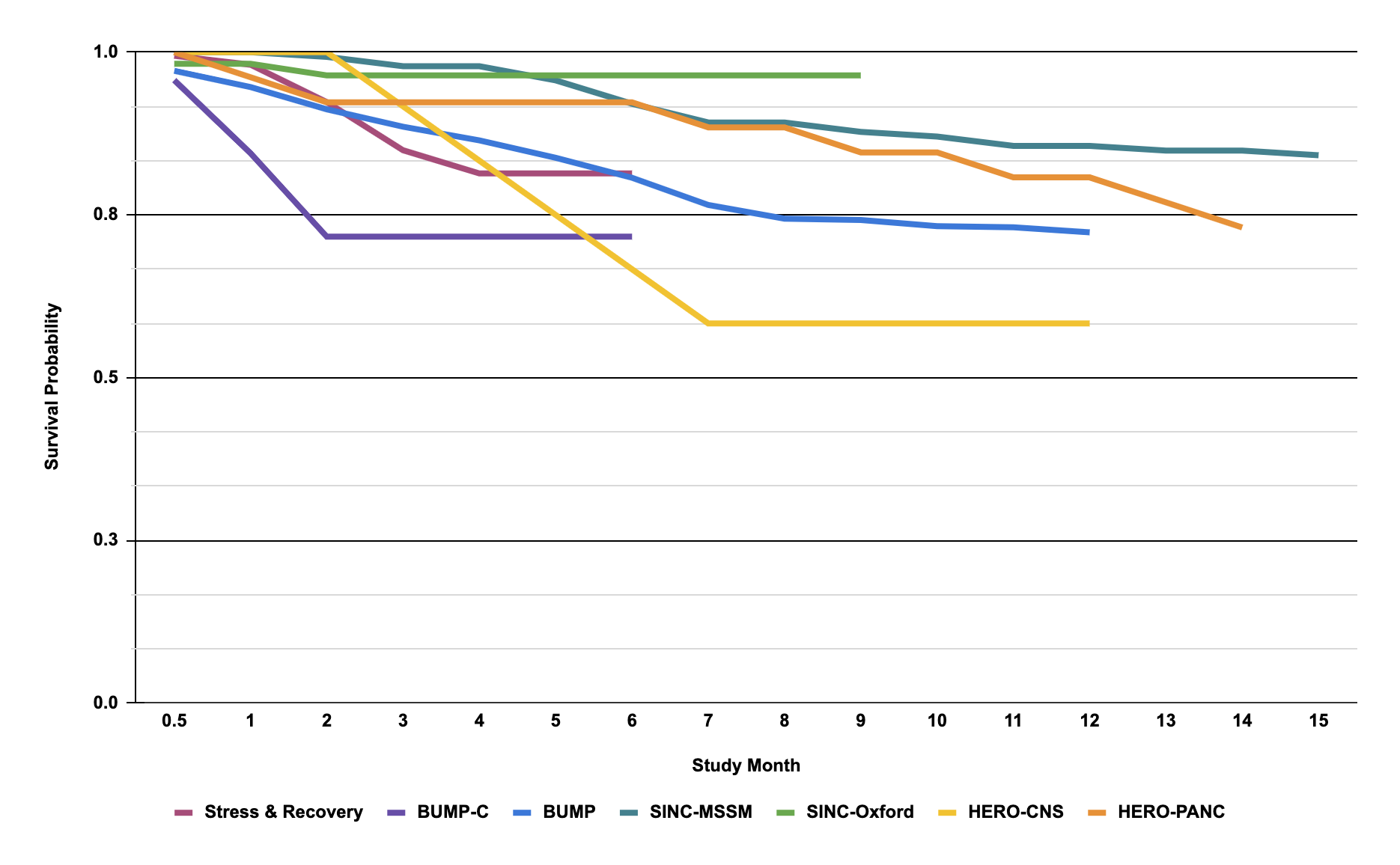

Supplement: Multimedia Appendix 4 [file jmir_v26i1e57827_app4.docx]
