# Supplementary material for: Value of Engagement in Digital Health Technology Research: Evidence Across 6 Unique Cohort Studies
Source: J Med Internet Res. 2024 Sep 3;26:e57827. doi: 10.2196/57827 (PMC11408887; doi:10.2196/57827)
Supplement: Multimedia Appendix 5 [file jmir_v26i1e57827_app5.docx]

|  | STRESS & RECOVERY  (N=297) | | | BUMP-C  (N=98) | | | BUMP-PRE  (N=379) | | | | | | SINC-MSSM  (N=117) | | | | | SINC-Oxford  (N=54) | | | |
| --- | --- | --- | --- | --- | --- | --- | --- | --- | --- | --- | --- | --- | --- | --- | --- | --- | --- | --- | --- | --- | --- |
|  | Check Ins | Oura | Daily Survey | Check Ins | Oura | Daily Survey | Check Ins | Oura | Garmin | Apple | Body Port | Daily Survey | Check Ins | Oura | Empatica | Body Port | "Daily" Survey | Check Ins | Oura | Empatica | Dail" Survey |
| Age (years) |  | | | | | | | | | | | | | | | | | | | | |
| 18 - 25 | 37 (75.0%) | 37 (92.9%) | 37 (73.1%) | 3 (90.5%) | 3 (94.9%) | 3 (19.1%) | 12 (91.3%) | 12 (63.1%) | 10 (76.0%) | 2 (62.5%) | 12 (40.5%) | 12 (38.3%) | 37 (50.0%) | 37 (70.3%) | 37 (10.4%) | 37 (25.8%) | 37 (21.2%) | 3 (100.0%) | 3 (81.3%) | 3 (3.1%) | 3 (23.8%) |
| 26 - 35 | 129 (80.0%) | 129 (97.6%) | 125 (75.9%) | 66 (100.0%) | 66 (90.6%) | 66 (42.4%) | 265 (100.0%) | 260 (87.8%) | 205 (96.1%) | 62 (98.6%) | 262 (73.4%) | 264 (61.9%) | 46 (59.8%) | 46 (82.6%) | 46 (37.4%) | 46 (39.1%) | 46 (28.3%) | 20 (100.0%) | 20 (94.8%) | 20 (48.7%) | 20 (60.3%) |
| 36 - 45 | 67 (66.7%) | 67 (95.2%) | 63 (76.8%) | 29 (100.0%) | 29 (88.8%) | 29 (45.7%) | 102 (100.0%) | 102 (87.5%) | 79 (98.0%) | 24 (98.2%) | 102 (78.4%) | 102 (59.7%) | 22 (47.7%) | 22 (75.4%) | 22 (19.3%) | 22 (48.1%) | 22 (32.7%) | 13 (100.0%) | 13 (99.6%) | 13 (76.2%) | 13 (79.0%) |
| 46+ | 64 (75.0%) | 63 (100.0%) | 61 (76.8%) | - | - | - | - | - | - | - | - | - | 12 (37.4%) | 12 (93.1%) | 12 (58.7%) | 12 (56.5%) | 12 (39.8%) | 18 (100.0%) | 18 (99.0%) | 18 (82.0%) | 18 (67.1%) |
| *P*-value* | <0.01 | 0.08 | 0.85 | 0.55 | 0.59 | 0.30 | 0.96 | 0.03 | 0.06 | 0.02 | 0.01 | 0.02 | 0.46 | 0.02 | 0.10 | 0.01 | 0.19 | 0.94 | 0.13 | 0.09 | 0.31 |
| Gender |  | | | | | | | | | | | | | | | | | | | | |
| Female | 264 (75.0%) | 263 (96.4%) | 254 (74.0%) | 98 (100.0%) | 98 (90.6%) | 98 (42.4%) | 379 (100.0%) | 374 (87.2%) | 294 (96.7%) | 88 (98.1%) | 376 (74.7%) | 378 (60.1%) | 74 (50.0%) | 74 (79.6%) | 74 (31.1%) | 74 (37.8%) | 74 (28.3%) | 29 (100.0%) | 29 (98.9%) | 29 (76.3%) | 29 (76.7%) |
| Male | 33 (75.0%) | 33 (97.3%) | 32 (79.0%) | - | - | - | - | - | - | - | - | - | 43 (44.4%) | 43 (84.9%) | 43 (23.2%) | 43 (38.5%) | 43 (27.9%) | 25 (100.0%) | 25 (98.7%) | 25 (63.6%) | 25 (54.8%) |
| *P*-value** | 0.99 | 0.69 | 0.60 | - | - | - | - | - | - | - | - | - | 0.35 | 0.39 | 1.00 | 0.56 | 0.70 | 0.56 | 0.76 | 0.56 | 0.02 |
| Race/Ethnicity |  | | | | | | | | | | | | | | | | | | | | |
| White | 242 (75.0%) | 241 (97.3%) | 234 (76.3%) | 76 (100.0%) | 76 (94.4%) | 76 (44.6%) | 299 (100.0%) | 296 (88.4%) | 232 (97.3%) | 69 (97.9%) | 296 (76.1%) | 298 (60.8%) | 99 (50.0%) | 99 (83.1%) | 99 (31.6%) | 99 (38.4%) | 99 (29.1%) | 51 (100.0%) | 51 (99.0%) | 51 (76.2%) | 51 (71.7%) |
| *White - Not Hispanic/Latino* | *237 (75.0%)* | *236 (97.3%)* | *229 (76.8%)* | - | - | - | *287 (100.0%)* | *284 (89.0%)* | *222 (97.8%)* | *67 (97.9%)* | *284 (76.4%)* | *286 (61.1%)* | *91 (50.0%)* | *91 (83.1%)* | *91 (31.6%)* | *91 (39.7%)* | *91 (30.0%)* | *49 (100.0%)* | *49 (99.0%)* | *49 (68.7%)* | *49 (68.8%)* |
| *White - Hispanic/Latino* | *5 (75.0%)* | *5 (97.1%)* | *5 (74.1%)* | - | - | - | *12 (94.0%)* | *12 (75.2%)* | *10 (93.0%)* | *2 (79.4%)* | *12 (51.0%)* | *12 (58.3%)* | *8 (36.7%)* | *8 (81.9%)* | *8 (30.7%)* | *8 (27.1%)* | *8 (20.2%)* | *2 (100.0%)* | *2 (98.5%)* | *2 (98.5%)* | *2 (84.8%)* |
| Black or African American | 8 (83.8%) | 8 (97.7%) | 7 (84.8%) | 8 (100.0%) | 8 (75.5%) | 8 (37.2%) | 13 (100.0%) | 12 (74.6%) | 9 (86.4%) | 4 (90.8%) | 13 (66.2%) | 13 (70.3%) | 4 (35.4%) | 4 (50.6%) | 4 (7.3%) | 4 (37.4%) | 4 (38.1%) | - | - | - | - |
| Asian / Pacific Islander | 22 (75.0%) | 22 (96.4%) | 20 (64.7%) | 5 (100.0%) | 5 (84.3%) | 5 (57.7%) | 32 (100.0%) | 31 (91.9%) | 26 (92.0%) | 7 (99.5%) | 32 (79.9%) | 32 (65.5%) | 5 (50.0%) | 5 (70.4%) | 5 (24.0%) | 5 (51.3%) | 5 (36.3%) | 1 (76.9%) | 1 (97.4%) | 1 (76.3%) | 1 (90.8%) |
| Other*** | 20 (61.3%) | 20 (95.8%) | 20 (66.8%) | 9 (66.7%) | 9 (53.4%) | 9 (19.7%) | 25 (92.3%) | 25 (79.6%) | 19 (95.7%) | 6 (96.7%) | 25 (65.5%) | 25 (51.1%) | 5 (50.0%) | 5 (80.5%) | 5 (12.7%) | 5 (42.7%) | 5 (10.4%) | 1 (75.0%) | 1 (97.3%) | 1 (35.7%) | 1 (29.9%) |
| Unknown / Not Reported | 5 (75.0%) | 5 (95.2%) | 5 (41.7%) | - | - | - | 10 (88.3%) | 10 (69.9%) | 8 (78.9%) | 2 (73.9%) | 10 (51.7%) | 10 (8.8%) | 4 (73.2%) | 4 (59.7%) | 4 (23.1%) | 4 (39.6%) | 4 (12.1%) | 1 (100.0%) | 1 (8.8%) | 1 (10.0%) | 1 (1.3%) |
| *P*-value* | 0.12 | 0.13 | 0.07 | <0.001 | 0.00 | 0.02 | 0.02 | 0.06 | 0.47 | 0.67 | 0.05 | <0.01 | 0.80 | 0.68 | 0.93 | 0.75 | 0.24 | 0.32 | 0.00 | 0.37 | 0.04 |

*ANOVA; **Mann-Whitney U Test; ***Native American or American Indian, Multi-Racial, Other

BUMP: Better Understanding the Metamorphosis of Pregnancy: BUMP-C: BUMP-Conception; SinC-MSSM: Stress in Crohn’s - Mount Sinai School of Medicine
