# Supplementary material for: Value of Engagement in Digital Health Technology Research: Evidence Across 6 Unique Cohort Studies
Source: J Med Internet Res. 2024 Sep 3;26:e57827. doi: 10.2196/57827 (PMC11408887; doi:10.2196/57827)
Supplement: Multimedia Appendix 6 [file jmir_v26i1e57827_app6.docx]

|  | Stress & Recovery  (N=365) | | | BUMP-C  (N=187) | | | BUMP  (N=524) | | | SINC-MSSM  (N=139) | | | SINC-Oxford  (N=56) | | |
| --- | --- | --- | --- | --- | --- | --- | --- | --- | --- | --- | --- | --- | --- | --- | --- |
|  | Retained  (n=297) | Not Retained  (n=68) | *P-value* | Retained  (n=98) | Not Retained  (n=53) | *P-value* | Retained  (n=379) | Not Retained  (n=145) | *P-value* | Retained  (n=117) | Not Retained  (n=22) | *P-value* | Retained  (n=54) | Not Retained  (n=2) | *P-value* |
| Age (years) |  | | | | | | | | | | | | | |  |
| 18 - 25 | 37 (12.5%) | 10 (14.7%) | 0.10* | 3 (3.1%) | 2 (3.8%) | 0.65** | 12 (3.2%) | 9 (6.2%) | 0.28* | 37 (31.6%) | 7(31.8%) | 1.00** | 3 (5.6%) | 1 (50.0%) | 0.08** |
| 26 - 35 | 129 (43.4%) | 39 (57.4%) |  | 66 (67.4%) | 39 (73.6%) |  | 265 (69.9%) | 98 (67.6%) |  | 46 (39.3%) | 9(40.9%) |  | 20 (37.0%) | 0 |  |
| 36 - 45 | 67 (22.6%) | 11 (16.2%) |  | 29 (29.6%) | 12 (22.6%) |  | 102 (26.9%) | 38 (26.2%) |  | 22 (18.8%) | 4(18.2%) |  | 13 (24.1%) | 0 |  |
| 46+ | 64 (21.6%) | 8 (11.8%) |  | -- | -- |  | -- | -- |  | 12 (10.3%) | 2(9.1%) |  | 18 (33.3%) | 1 (50.0%) |  |
| Gender |  | | | | | | | | | | | | | |  |
| Female | 264 (88.9%) | 61 (89.7%) | 1.00* | 98 (100.0%) | 53 (100.0%) | -- | 379 (100.0%) | 145 (100.0%) | -- | 74 (63.3%) | 16 (72.8%) | 0.54* | 29 (53.7%) | 2 (100.0%) | 0.50** |
| Male | 33 (11.1%) | 7 (10.3%) |  | 0 | 0 |  | 0 | 0 |  | 43 (36.8%) | 6 (27.3%) |  | 25 (46.3%) | 0 |  |
| Unknown / Not Reported | 0 | 0 |  | 0 | 0 |  | 0 | 0 |  | 0 | 0 |  | 0 | 0 |  |
| Race/Ethnicity |  | | | | | | | | | | | | | |  |
| White | 242 (81.5%) | 60 (88.2%) | 0.44** | 76 (77.6%) | 31 (58.5%) | <0.0001** | 299 (78.9%) | 98 (67.6%) | <0.001* | 99 (84.6%) | 20 (90.9%) | 0.76** | 51 (94.4%) | 1 (50.0%) | -- |
| *White - Not Hispanic/Latino* | *237 (79.8%)* | *57 (83.8%)* |  |  |  |  | *287 (75.7%)* | *91 (62.7%)* |  | *91 (77.8%)* | *19 (86.4%)* |  | *49 (90.7%)* | *1 (50.0%)* |  |
| *White - Hispanic/Latino* | *5 (1.69%)* | *3 (4.4%)* |  |  |  |  | *12 (3.2%)* | *7 (4.8%)* |  | *8 (6.8%)* | *1 (4.6%)* |  | *2 (3.7%)* | *0* |  |
| Black or African American | 8 (2.7%) | 0 (0.0%) |  | 8 (8.2%) | 4 (7.6%) |  | 13 (3.4%) | 8 (5.5%) |  | 4 (3.4%) | 1 (4.6%) |  | 0 | 0 |  |
| Asian / Pacific Islander | 22 (7.4%) | 5 (7.4%) |  | 5 (5.1%) | 2 (3.8%) |  | 32 (8.4%) | 12 (8.3%) |  | 5 (4.3%) | 0 |  | 1 (1.9%) | 0 |  |
| Other*** | 20 (6.7%) | 3 (4.4%) |  | 9 (9.2%) | 2 (3.8%) |  | 25 (6.6%) | 11 (7.6%) |  | 5 (4.3%) | 1 (4.6%) |  | 1 (1.9%) | 0 |  |
| Unknown / Not Reported | 5 (1.7%) | 0 |  | 0 | 14 (26.4%) |  | 10 (2.6%) | 16 (11.0%) |  | 4 (3.4%) | 0 |  | 1 (1.9%) | 1 (50.0%) |  |

*Chi-square test; **Fisher’s exact test; *******Native American or American Indian, Multi-Racial, Other

BUMP: Better Understanding the Metamorphosis of Pregnancy: BUMP-C: BUMP-Conception; SinC-MSSM: Stress in Crohn’s - Mount Sinai School of Medicine
