# Supplementary material for: Value of Engagement in Digital Health Technology Research: Evidence Across 6 Unique Cohort Studies
Source: J Med Internet Res. 2024 Sep 3;26:e57827. doi: 10.2196/57827 (PMC11408887; doi:10.2196/57827)
Supplement: Multimedia Appendix 7 [file jmir_v26i1e57827_app7.docx]

*Participation burden and forgetfulness*

Common themes that arose across all included studies was the want for custom notifications. When collecting real-world data in remote settings it is impossible to find a consistent notification time to complete surveys that works for all participants. In light of this feedback, the study investigators altered these notifications eventually to enable the participant to be able to set their own custom notification time and this was received well across many of the included studies.

Participants commonly reported a want to know when tasks expired so that they could plan accordingly. Many participants indicated that they often missed tasks when finally finding time to complete without this knowledge. Potential solutions to this challenge include adding task expiry information into the app, and carefully weighing task expiry time versus key data collection windows.

App activity fatigue was observed among many participants, particularly among those who were not experiencing frequent symptoms or whose symptoms did not change over study follow-up. The study investigators included screening items across many symptom measures where participants can indicate no change, or no symptoms as opposed to having to complete an entire symptom measure which alleviated this burden in some. However, when completing repeated, high frequency tasks, task fatigue is an inevitability. An alternate solution is including a dynamic app schedule that changes on the basis symptom presence so that those with no change, or no symptoms are prompted to complete tasks at lower frequencies.

Many participants indicated challenges with cognitive active tasks that, while intended to be challenging as a measure of cognitive function, produced frustration and even embarrassment in some participants who worried about their performance.

*Physical and Mental Barriers*

Environmental barriers, such as workplace/occupational restrictions, size of home, outside weather made certain active tasks such as those that require participants to walk a certain distance in a straight line difficult. Providing participants with clear task instructions including ample alternative solutions to completing active tasks when these environmental barriers occur is a potential solution to this challenge. Particularly among the studies of older, more chronically ill participants (HERO studies), health-related barriers to task completion were observed, and certain symptoms (e.g., motor problems) created challenges in completing some of the active tasks for example. Other scenarios such as hospitalization, bed rest, or other acute illness cause barriers in longitudinal remote data capture. This was similarly observed among participants post delivery during the postpartum period. While an adherence loss is expected during periods of acute illness, or life changes such as caring for a newborn, this results in periods of missing data and without labeling these periods, it's unclear why this information is missing at the analysis phase. Embedding optional in-app questions that enable participants to report that they are not able to complete tasks is a useful feature so that the type of data missingness can be quantified. This also enables the use of missing data as a more informative potential feature in data analyses.

Numerous tech issues arose across all of the included studies relating to general logistics that disrupted participant experience, and in turn adherence during certain periods of time. Among studies that used the Oura smart ring, ensuring the correct ring size can be challenging (even after sending participants ring sizing kits). Researchers should expect a delay in study start among a proportion of participants owing to this challenge. Further, some participants had to switch out ring sizes due to weight changes. Different smartphone versions will invariably create troubleshooting issues among participants where some tasks look different, or the apps do not work in their intended way. Extensive beta testing across several different phone versions prior to study implementation can help with this, but there are invariably new issues that will arise during active study follow-up. A common challenge encountered when attempting to onboard participants in-clinic was poor wifi connectivity, resulting in many participants (for example from the HERO studies and Stress in Crohn’s) to set up their devices at home. Budgeting for wifi amplifiers could help with this study logistics challenge, or ensuring the location of study onboarding has sufficient strength. App bugs are a reality in many of these studies, and it is challenging to foresee all potential errors that may arise when implementing in a remote setting.

*Personal and altruistic benefit*

The engagement specialist calls across these studies served an important purpose of providing multiple opportunities to explain and re-iterate to participants why certain tasks were asked of them, and how certain passive features could be used to better understand their health and that of others. There were numerous questions about the nature of the objective wearable data, how to interpret it (e.g., how is heart rate variability related to my condition?) and why certain features were tracked semi-continuously. In turn, participants who had less of an opportunity to discuss questions with engagement specialists who had lower adherence on the check-in support calls showed lower adherence on study-related activities.

Many participants expressed that certain tasks or device data did not feel relevant to them and when this occurred, lower adherence was observed. This was sometimes the result of participants not experiencing symptoms and not understanding why they had to complete daily surveys. As opposed to selecting the ‘none’ category on these types of surveys, it was observed that some participants skipped them all together resulting in missing data. Explaining to participants the importance of being aware of absence of symptoms, and modifying the in-app surveys so that an initial screening question could be responded to as opposed to having to select ‘none’ for several items helped with this challenge.

Many participants did not fully understand why certain features were being tracked and in these instances lower adherence was observed for these specific tasks. For example, despite explanations provided in the consent form, participants struggled with understanding the utility of tracking phone usage and in particular, screen time. During engagement specialist calls, and participant-investigator Zoom calls (where applicable), it was attempted to provide more in depth explanations as to how this information could be used to better understand individuals health. For example, changes in phone usage could be used as an indicator of mood status.

*Digital Literacy*

Digital literacy was more of an issue among studies with older, more clinically unwell participants (e.g., HERO studies). While all of these studies included detailed participant guides with screenshots of how to onboard, sync each study device, troubleshoot common problems, and had research staff assist either in-clinic or over the phone, there remained feedback from some participants on challenges in getting started and working through tech issues. The studies described here included considerable support for onboarding compared to many existing digital health studies that rely on participants to do this themselves remotely. The fact that this theme still arises even among highly participant centric and engaged studies signifies the complexity of implementing wearable based studies that require syncing of multiple devices and daily interactions with an app to sync data for example. A study video that walks participants through this process could be valuable in alleviating this issue, while trying to onboard participants in person alleviates this challenge, particularly for older populations.

Feedback from many participants irrespective of age was the want for more education and knowledge around the returned device data. Many consumer-grade wearable devices return objectively measured data back to participants, such as heart rate variability, sleep quality and activity metrics. Some participants expressed challenges in interpreting what these values indicate. This is not a digital literacy challenge per se, as many of the objective measures of health captured from wearable-based devices are indeed challenging to interpret as they are not yet validated, nor is it fully understood how they may relate to different health states. The study investigators attempted to alleviate this challenge by providing participants with information on device readout definitions, and hypothesized relationships with different conditions which was appreciated by many.

*Privacy and Confidentiality*

Across all of the included studies, the participant centric engagement approaches include strong emphasis on working with participants in transparent ways so that they fully understand the use of their data. Privacy and Confidentiality concerns were not a major hurdle across these studies. However, certain types of data, and 3rd party apps were conveyed as concerning. These are related to the video diaries that include the collection of face and audio data, and 3rd party apps such as RescueTime that passively collect screen time and other phone use metrics. Additionally when integrating with other 3rd party device companies, participants will have to offer personal information (as they would when engaging with these apps outside research). Allowing participants to opt out of certain data streams that are not absolutely essential to the primary research objective can help with this. Additionally, limiting passive data captured to metadata that only includes summary metrics such as number of texts or social media posts, as opposed to the content (words) can reduce these intrusive data collection concerns.
